# The Karlsruhe Metabolomics and Nutrition (KarMeN) Study: Protocol and Methods of a Cross-Sectional Study to Characterize the Metabolome of Healthy Men and Women

**DOI:** 10.2196/resprot.5792

**Published:** 2016-07-15

**Authors:** Achim Bub, Anita Kriebel, Claudia Dörr, Susanne Bandt, Manuela Rist, Alexander Roth, Eva Hummel, Sabine Kulling, Ingrid Hoffmann, Bernhard Watzl

**Affiliations:** ^1^ Max Rubner-Institut Department of Physiology and Biochemistry of Nutrition Karlsruhe Germany; ^2^ Max Rubner-Institut Department of Nutritional Behaviour Karlsruhe Germany; ^3^ Max Rubner-Institut Department of Safety and Quality of Fruit and Vegetables Karlsruhe Germany

**Keywords:** metabolomics, metabolome, humans, healthy subjects, cross-sectional studies

## Abstract

**Background:**

The human metabolome is influenced by various intrinsic and extrinsic factors. A precondition to identify such biomarkers is the comprehensive understanding of the composition and variability of the metabolome of healthy humans. Sample handling aspects have an important impact on the composition of the metabolome; therefore, it is crucial for any metabolomics study to standardize protocols on sample collection, preanalytical sample handling, storage, and analytics to keep the nonbiological variability as low as possible.

**Objective:**

The main objective of the KarMeN study is to analyze the human metabolome in blood and urine by targeted and untargeted metabolite profiling (gas chromatography-mass spectrometry [GC-MS], GC×GC-MS, liquid chromatography-mass spectrometry [LC-MS/MS], and^1^H nuclear magnetic resonance [NMR] spectroscopy) and to determine the impact of sex, age, body composition, diet, and physical activity on metabolite profiles of healthy women and men. Here, we report the outline of the study protocol with special regard to all aspects that should be considered in studies applying metabolomics.

**Methods:**

Healthy men and women, aged 18 years or older, were recruited. In addition to a number of anthropometric (height, weight, body mass index, waist circumference, body composition), clinical (blood pressure, electrocardiogram, blood and urine clinical chemistry) and functional parameters (lung function, arterial stiffness), resting metabolic rate, physical activity, fitness, and dietary intake were assessed, and 24-hour urine, fasting spot urine, and plasma samples were collected. Standard operating procedures were established for all steps of the study design. Using different analytical techniques (LC-MS, GC×GC-MS,^1^H NMR spectroscopy), metabolite profiles of urine and plasma were determined. Data will be analyzed using univariate and multivariate as well as predictive modeling methods.

**Results:**

The project was funded in 2011 and enrollment was carried out between March 2012 and July 2013. A total of 301 volunteers were eligible to participate in the study. Metabolite profiling of plasma and urine samples has been completed and data analysis is currently underway.

**Conclusions:**

We established the KarMeN study applying a broad set of clinical and physiological examinations with a high degree of standardization. Our experimental approach of combining scheduled timing of examinations and sampling with the multiplatform approach (GC×GC-MS, GC-MS, LC-MS/MS, and^1^H NMR spectroscopy) will enable us to differentiate between current and long-term effects of diet and physical activity on metabolite profiles, while enabling us at the same time to consider confounders such as age and sex in the KarMeN study.

**Trial Registration:**

German Clinical Trials Register DRKS00004890; https://drks-neu.uniklinik-freiburg.de/drks_web/navigate.do? navigationId=trial.HTML&TRIAL_ID=DRKS00004890 (Archived by WebCite at http://www.webcitation.org/6iyM8dMtx)

## Introduction

The metabolome represents the complete set of small molecules or metabolites in a biological system, which in the case of blood and urine provides valuable information on human metabolism. The most frequently used analytical techniques for the identification and quantification of metabolites are nuclear magnetic resonance (NMR) spectroscopy, liquid chromatography-mass spectrometry (LC-MS), and gas chromatography-mass spectrometry (GC-MS). During the last decades, analytical techniques have significantly progressed allowing the measurement of hundreds of metabolites in a single sample. Today, the metabolomics approach is an important tool in biomedical research, which contributes to the identification of new potential biomarkers of diseases. Specific metabolites and metabolite patterns have been linked to various diseases, such as cardiovascular disease [1-3], cancer [4-7], and many others [8,9]. Further, metabolomics is also a promising approach in nutrition research [10-12]. It allows studying the association between human metabolism and diet more comprehensively compared to classical analytical methods. In addition, it may complement current methods of assessing dietary intake.

The human metabolome is characterized by a large variability. Endogenous and exogenous factors have been described to influence metabolic profiles. Examples of endogenous or subject-related factors are age [13-16], sex [17-20], body composition [21-23], hormone status (eg, menstrual cycle, menopause) [16,24,25], circadian rhythm [26-29], and physical fitness [30,31]. Among exogenous and lifestyle factors, smoking [32], alcohol intake [33,34], diet [35-37], and physical activity and exercise [38-40] have been addressed so far. In addition to these subject-related analytical issues, preanalytical aspects have also been identified to contribute to metabolome variability. These include sample and data collection, sample preparation, and storage issues [41-46].

Recently, the composition of the human metabolome has been reported for plasma [47], serum [48,49], and urine [50,51]. However, these studies primarily focused on expanding the knowledge of the entire human metabolome and thus did not consider age, sex, and other factors as contributors to the variability of the human metabolome [48,49,51].

Combining targeted and nontargeted NMR spectroscopy, GC-MS, and LC-MS methods may be favorable in identifying a broad spectrum of metabolites from a single sample compared to the application of only one metabolomics platform. Unfortunately, these procedures have not been applied in a single, but in different, sample sets [48], or have been applied in a small group of volunteers only [51]. Additionally, samples for metabolite profiling may be purchased from companies [49] with volunteer information totally missing.

Most studies assessing the composition of the human metabolome did not consider the preceding given factors, such as age, sex, body composition, diet, physical activity, and fitness, which may impact the human metabolome. Although some studies investigated the influence of factors such as age [13-16] on metabolite profiles, they did not report information on diet or physical activity. Similarly, studies investigating the impact of physical activity did not consider the role of sex [38,39].

Overall, there is currently no comprehensive consideration and understanding of the major endogenous and exogenous determinants of the human metabolome, such as age, sex, body composition, diet, physical activity, and fitness. A combined targeted and untargeted metabolite profiling (GC×GC-MS, GC-MS, LC-MS/MS, and^1^H NMR spectroscopy) of plasma and urine in a well-characterized population is missing. Therefore, we performed the Karlsruhe Metabolomics and Nutrition (KarMeN) study, in which we established a strictly scheduled experimental setting with a high degree of experimental standardization in order to minimize variations regarding examination, sample handling, and analysis. Based on standard operating procedures (SOPs), that were applied on recruitment, examinations, and on the preanalytical handling, all participants underwent the same procedures according to a specified timeline and all samples were treated identically.

The main objective of the KarMeN study is to analyze the human metabolome in blood and urine by targeted and untargeted metabolite profiling (GC×GC-MS, GC-MS, LC-MS/MS, and^1^H NMR spectroscopy) and to determine the impact of sex, age, body composition, diet, and physical activity on metabolite profiles of healthy women and men. Here, we report the outline of the study protocol. Details on clinical measurements will be published elsewhere, whereas the description of analytical procedures has already been published [52,53].

## Methods

### Study Design and Setting

**Figure 1:**
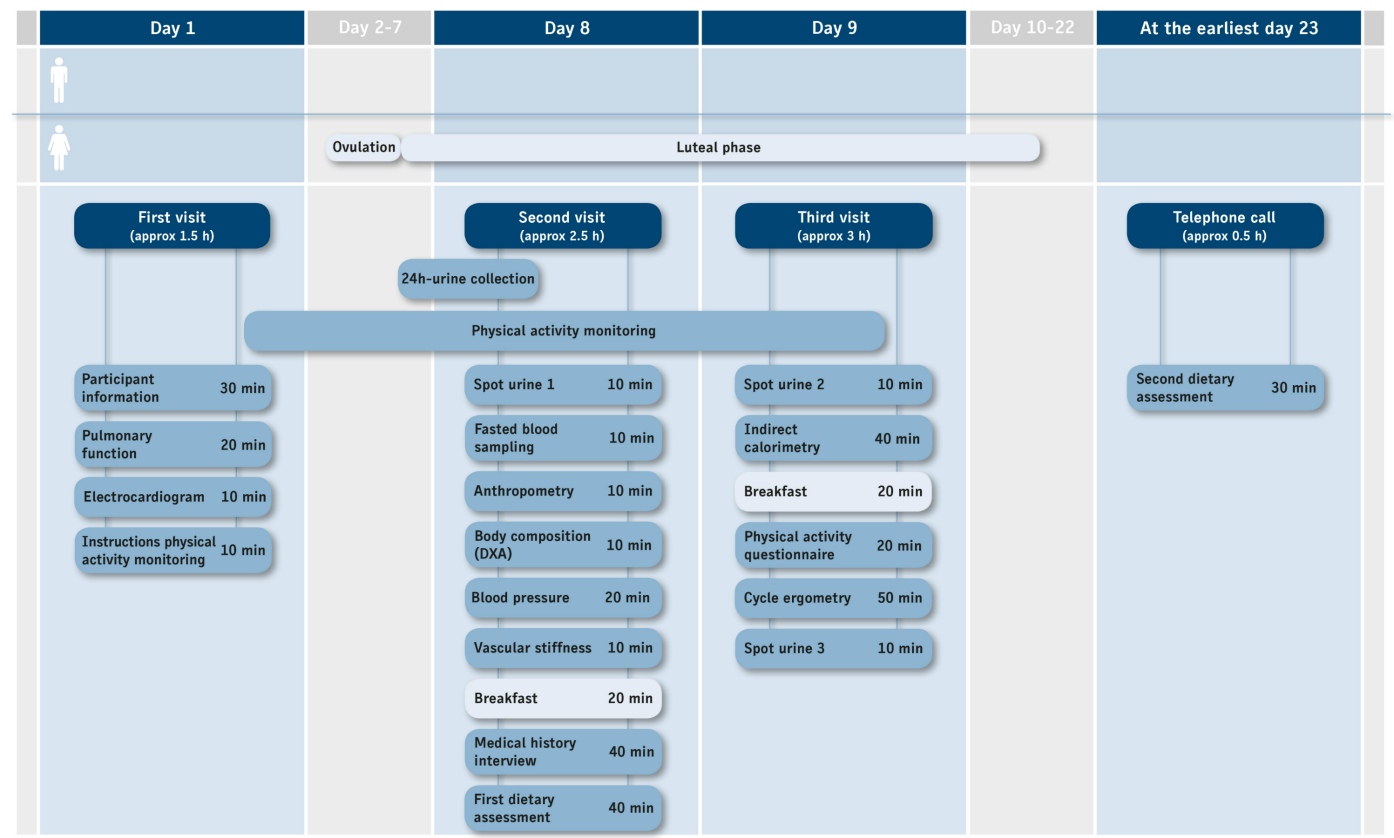
Overview of KarMeN study days and examination schedule.

The KarMeN study was performed at the Division of Human Studies of the Max Rubner-Institut in Karlsruhe, Germany, between March 2012 and July 2013. All volunteers visited the study center within a period of 9 days for a total of three visits, in which the second visit was scheduled exactly 1 week after the first, and the third visit was on the day following the second visit (Figure 1). Participants underwent a series of examinations and followed an identical schedule on each of the three examination days (Figure 1). SOPs were developed for any procedure related to examinations, measurements, sample collection, sample preparation, and storage. Examiners were trained before, and were supervised during, recruitment.

### Participants

Healthy, nonsmoking volunteers older than 18 years of age were eligible to participate. Detailed inclusion and exclusion criteria are listed in Textbox 1. Health status examination included the assessment of medical history and a basic physical examination. Women taking hormonal contraceptives or receiving hormonal replacement therapy were not included because of the significant impact of hormone intake on various aspects of human metabolism and physiology. As the phase of the menstrual cycle is known to impact the metabolite profile in women [25], premenopausal women were asked to document their regular menstrual cycle over a period of 3 months. Based on this information, women were scheduled for examinations within their luteal phase. During the anticipated phase before the examinations, they performed an ovulation test (OvuQuick, NanoRepro AG, Marburg, Germany) to verify ovulation and subsequent luteal phase. The status of postmenopausal women was determined by respective anamnestic interview and follicle-stimulating hormone (FSH) measurements (MZV Labor PD Dr Volkmann, Karlsruhe, Germany). We defined postmenopausal status by absence of menstrual bleeding for at least 1 year and FSH >25 IU/L.

**Inclusion and exclusion criteria for participants of the KarMeN study.**

**Inclusion criteria**

- Healthy men and women
- 18 years of age or older
- Nonsmokers
- Volunteers conducting all examinations and tests
- Participants giving their written and informed consent

**Exclusion criteria**

- Smokers
- Volunteers on regular medication
- Volunteers taking supplements
- Women using hormonal contraceptives
- Pregnant or breastfeeding women
- Volunteers with diseases of the cardiovascular system, lungs, gastrointestinal tract, metabolism, skin, viscera, nervous system, and infectious or immunological diseases in therapeutic need
- Volunteers with known allergy against *para*-aminobenzoic acid (PABA)
- Volunteers with intolerance against Finalgon
- Volunteers with tumors
- Volunteers with acute or chronic infectious diseases
- Volunteers with drug or alcohol abuse
- Volunteers who may not adhere to the study protocol
- Volunteers who gave no written consent
- Institutionalized patients in psychiatric hospitals

**Figure 2:**
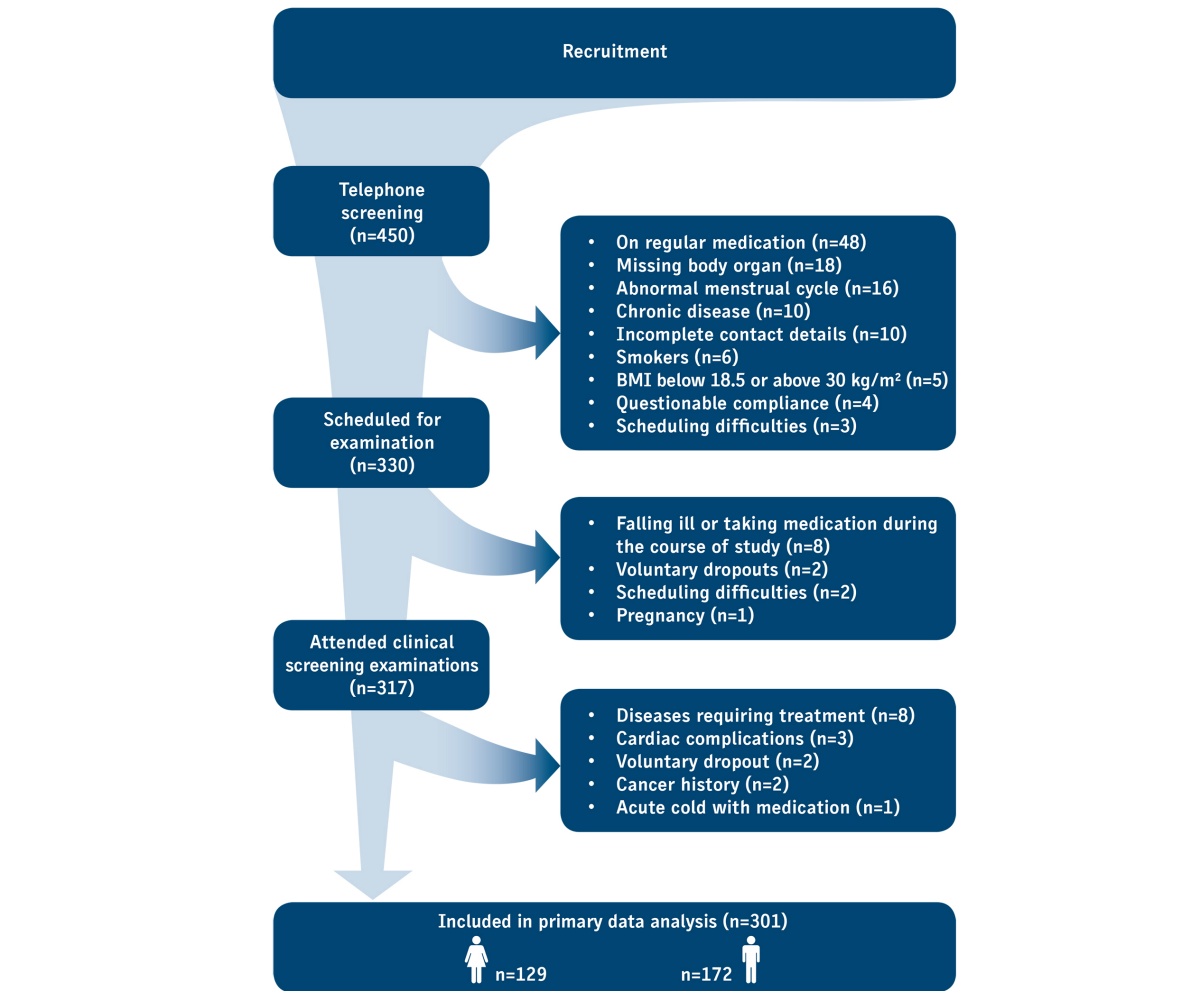
Flowchart describing the recruitment process of the KarMeN study. Along the vertical arrow, the phases of recruitment are shown and the number of volunteers entering each phase. On the right hand side, reasons for exclusion at each stage are given.

Recruitment procedures included direct communication with previous study participants, advertisements in local media (newspapers and radio), flyers, and word of mouth. Initially 450 persons contacted the study center (Figure 2). Those who passed an initial telephone screening done with a checklist were scheduled for a medical examination (n=330). Reasons for exclusion of participants are given in Textbox 1.

### Sample Collection

Blood samples were collected after at least 10 hours fasting for the clinical blood profile and hormonal and metabolome analysis. Volunteers were placed on an examination couch in the supine position and blood samples were obtained by an experienced study nurse from an antecubital vein. After cutaneous disinfection, a tourniquet was moderately applied before venipuncture (safety blood collection device: Venofix Safety, Braun AG, Melsungen, Germany). Blood was drawn into S-Monovette tubes (Sarstedt AG & Co, Nümbrecht, Germany). Plasma was obtained by using the EDTA S-Monovette, serum by Serum-Gel S-Monovette; for immunological assays, the Lithium-Heparin S-Monovette was used. After venipuncture, blood- containing ethylenediaminetetraacetic acid (EDTA) tubes for plasma preparation were immediately placed on ice before centrifugation (CR 4.22, Jouan, Saint-Herblain, France).

Volunteers collected urine from the morning of the day before blood donation until the morning of the day of blood donation at visit 2 (24-hour urine collection). Completeness of the 24-hour urine was checked with the *para*-aminobenzoic acid (PABA) method [54]. For 24-hour urine collection, volunteers were instructed by a study nurse and received a protocol with written instructions and for documentation. They were provided with two urine containers (volume 2 liters, Sarstedt AG & Co, Nümbrecht, Germany) and were asked to store the urine containers at home in the refrigerator and to place them in a thermal bag containing prechilled thermal packs for transport to the study center. In addition, volunteers provided fasting spot urine at the study center on two consecutive days (visits 2 and 3) (Figure 1). Additional spot urine was collected after cycle ergometry (visit 3). Spot urine samples were collected in 100 mL polypropylene collection cups with screw caps (Sarstedt AG & Co, Nümbrecht, Germany) and immediately placed on ice for a maximum 10 minutes before further processing. Material for sample collection and storage were derived from a single production batch.

### Sample Processing and Storage

**Figure 3:**
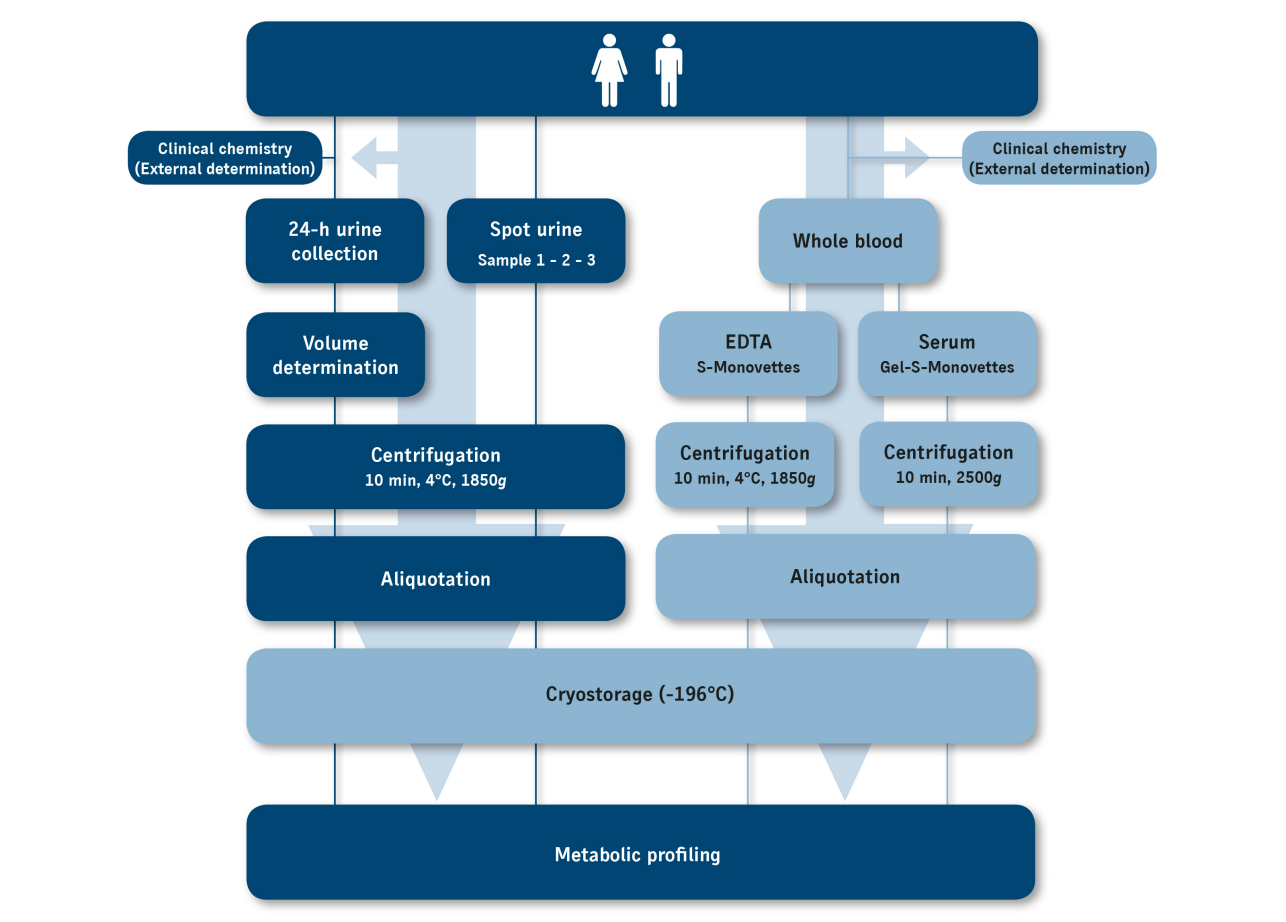
Schematic representation of the procedures for preanalytical sample handling. After collection, standard parameters in blood and 24-hour urine were analyzed by a certified external clinical chemistry laboratory. All other samples were identically processed at the study center based on a given schedule with defined time intervals between the different preparation steps of centrifugation and aliquotation until cryostorage.

After collection, samples were processed identically based on a given schedule with defined time intervals between the different preparation steps (see Figure 3). For plasma preparation, EDTA blood was centrifuged (4°C, 10 min, 1850 *g*, CR 4.22, Jouan, Saint-Herblain, France) and supernatants were combined before aliquoting. Serum was obtained after blood clotting for 30 minutes at room temperature and centrifugation (room temperature, 10 min, 2500 *g*). Urine samples were centrifuged at 1850 *g* (4°C, 10 min) to remove cellular particles and debris [55]. Before processing, all urine samples were checked with urine test strips (CombiScreen, CombiScan 100, Analyticon) to exclude samples with pathological aspects.

All samples were transferred into prechilled cryovials (Brand, Germany) by means of electronically regulated pipettes (Eppendorf Multipette Stream, Germany) using disposable tips (Eppendorf Combitips, Germany). Cooling of cryovials during partitioning was achieved by placing them into cooled (–80°C) aluminum racks. For practical handling reasons, samples from each individual generated at visits 2 and 3 were stored in the laboratory at –20°C for up to 2 days and then transferred into the gas phase of the liquid nitrogen (LN_2_) storage system until analysis. This procedure has previously been shown not to affect metabolomics results [45].

### Examinations and Outcome Measurements

Examinations and measurements included anthropometric parameters (weight, height, waist circumference), body composition by dual-energy x-ray absorptiometry (DXA; Lunar iDXA, GE Healthcare, Germany), pulmonary function (FlowScreen, CareFusion, Hoechberg, Germany), electrocardiogram (ECG; Cardioline AR1200, Cavareno, Italy), blood pressure (Boso Carat Professional, Bosch & Sohn, Jungingen, Germany), and arterial stiffness (ARTERIOGraph, Medexpert, Budapest, Hungary).

Food consumption for the day before blood sample drawing and the day of 24-hour urine collection was assessed by the 24-hour recall method using the software EPIC-Soft [56]. In order to differentiate between the impact of acute and long-term diet on the human metabolome, a second 24-hour recall was conducted by telephone at least 2 weeks after the first interview. Additionally, a food frequency questionnaire developed specifically for this study was conducted. It covered food consumption for the last year. Nutrient intake was calculated based on the German Nutrient Database (BLS) version 3.02 [57]. Supplement use was not assessed because participants with supplement use were excluded from the study. To calculate long-term food consumption and long-term nutrient intake, the Multiple Source Method [58,59] was applied.

Physical activity was assessed for the day before blood sampling and for an average of the study week by combined accelerometry and heart rate measurements (Actiheart, CamNtech, Cambridge, UK). An average of the weekly physical activity for the last 3 months was determined by the standardized International Physical Activity Questionnaire (IPAQ) [60]. Cardiorespiratory fitness was determined by cycle ergometry with combined capillary lactate measurements. Details on physical activity and physical fitness methods will be described elsewhere. Additionally, basal metabolic rate was determined by indirect calorimetry (Vmax Encore, CareFusion, Hoechberg, Germany). During visits at days 2 and 3, volunteers received a breakfast after their examinations. At visit 2, breakfast was given ad libitum, whereas at visit 3, breakfast was adjusted to individual energy requirements and was provided approximately 45 minutes before cycle ergometry. Examinations, blood sampling, 24-hour urine, and spot urine collection were arranged in this setting to ideally combine data from volunteer examinations, food consumption, and physical activity measurement with analytical data from blood and urine metabolite profiling (Figures 1 and 2). Blood and 24-hour urine were analyzed for standard parameters by a certified clinical chemistry laboratory (MZV Labor PD Dr Volkmann, Karlsruhe, Germany).

### Metabolomics Analyses

Complementary analytical methods and techniques for targeted and untargeted metabolite profiling of biofluids (GC×GC-MS, GC-MS, LC-MS/MS, and^1^H NMR spectroscopy) were applied. All 24-hour urine and fasted plasma samples were analyzed by untargeted GC×GC-MS using a Shimadzu GCMS QP2010 Ultra instrument equipped with a ZOEX ZX2 modulator according to the method established previously [52]. With this method, a wide range of metabolites can be detected, such as amines, amino acids, organic acids, sugars, sugar alcohols, other polyols, etc. Because some isomeric sugar species cannot be sufficiently resolved with the untargeted GC×GC-MS approach, but may play an important role in human metabolism, a complementary targeted GC-MS sugar profiling method was developed for urine samples using a Shimadzu GCMS QP2010 Ultra instrument. Overall, 66 metabolites, consisting of 38 known sugar species, 17 unknown sugar species, and 11 nonsugar compounds, were detected with this method. The chromatographic separation of plasma fatty acids usually requires the application of specialized polar columns and, thus, cannot be done adequately using a standard apolar × medium-polar GC×GC column setup. For this reason, we used a targeted GC-MS analysis method [61] to determine plasma fatty acids as methyl esters, with minor modifications. Using a GC single quadrupole instrument (Shimadzu GCMS QP2010 Ultra) and a BPX90 column (Trajan Scientific), 48 fatty acids could be determined in plasma. LC-MS metabolite profiling using the Absolute IDQ p180 kit developed by Biocrates AG (Innsbruck, Austria) was applied to determine acyl carnitines, amino acids, biogenic amines, phosphatidylcholines, and sphingomyelins in fasted plasma samples. A targeted quantification UPLC-MS/MS method for seven amino compounds in plasma, including L-carnitine, choline, and trimethylamine *N*-oxide (TMAO), was established using an Acquity UPLC H-Class system coupled to a Xevo TQD triple quadrupole MS (both from Waters, Eschborn, Germany). Targeted LC-MS analysis of 14 bile acids was done from fasted plasma using a 1200 series HPLC system (Agilent, Waldbronn, Germany) coupled to a Q-Trap 3200 mass spectrometer (AB Sciex, Darmstadt, Germany) as described elsewhere [62]. All plasma and urine samples were analyzed by untargeted one-dimensional^1^H NMR spectroscopy [45]. Typically, metabolites that can be detected include organic acids, amino acids, amines, sugars, sugar alcohols, and others.

### Data Processing

The GC×GC-MS raw data files were processed by untargeted alignment by in-house-developed R-modules, as described previously [53]. Signal intensity drift (ie, intra- and interbatch effects) occurring during the 4- to 5-week measurement period were corrected by means of regularly injected quality control (QC) samples. For the data of the semitargeted GC-MS analysis of sugar species in urine, an automatic method for integration was prepared using the Post-run Analysis feature of GCMSSolution (v 4.1.1.). An MS Excel table with integrated peak areas of the chosen substances was made for further data processing.

To analyze the samples of the entire study by LC-MS metabolite profiling *(* Absolute IDQ p180 kit), five Absolute IDQ well plates were used. To account for possible batch effects between the plates, data normalization as described by the manufacturer’s user manual was applied based on the pooled QC samples, which were extracted and measured 10 times on each well plate in-between the study samples.

All NMR spectra were automatically phased with the Bruker AU program apk0.noe without referencing. Using the program AMIX (v 3.9.14.; Bruker, Rheinstetten, Germany) plasma spectra were then referenced to the EDTA signal at 2.5809 ppm and bucketed graphically, such that buckets wherever possible contained only one signal or group of signals and no peaks were split between buckets. Urine spectra were resampled to bring them to a uniform frequency axis. Then, spectra were aligned by “correlation optimized warping” [63] and bucketed using an in-house-developed software based on Python, again trying to define buckets that contain only one signal or group of signals and not splitting peaks between buckets whenever possible. Identification of important metabolites was achieved with Chenomx NMR Suite 8.1 (Chenomx, Edmonton, AB, Canada).

### Metabolomic Data Analysis and Statistics

Data for the different analytical platforms were integrated into a common data matrix, consisting of 301 samples and more than 1000 analytes (including knowns and unknowns). Analytes with a detected frequency lower than 75% in the study samples were eliminated from the data matrix before statistical analysis. In the final analysis, 442 plasma analytes and 531 urine analytes were included.

The columns of this common data matrix were mean centered and scaled by standard deviation before analysis. This resulting matrix was used as input for three different prediction models (support vector machine with linear kernel, generalized linear model, and partial least squares). When the model is used to predict categorially considered variables, the classification accuracy is assessed. For continuous outcomes, the parameters root mean squared error and *R*^2^ are calculated to estimate performance of the predictions. Furthermore, multivariate linear regression models are calculated with standard variables of interest including sex, age, and anthropometric variables. Finally, a ranking of the top metabolites with regard to accordance in the metabolite patterns is created. Therefore, we calculate a rank for every metabolite for each algorithm. For the final ranking, the ranks of categorially considered variables are averaged.

### Ethics and Dissemination

The study has been performed in accordance with the Declaration of Helsinki. It was registered at the German Clinical Trials Register (No: DRKS00004890) and approved by the Ethics Committee of the State Medical Chamber of Baden-Württemberg, Stuttgart, Germany (F-2011-051). Approval for DXA measurements in healthy participants was obtained from the Federal Office for Radiation Protection (Z5-22462/2-2011-043). All participants were informed in detail about the examinations, procedures, and measurements, and gave their written consent. Confidentiality of personal data is guaranteed by following the regulations of the State Medical Chamber and the Federal Data Protection Act. Access to data is restricted to project partners, who receive only coded data for analysis.

## Results

The project was funded in 2011 and enrollment was carried out between March 2012 and July 2013. Finally, a total of 301 volunteers were eligible to participate in the study and primary analysis. Basic characteristics of the KarMeN study participants are given in Table 1. Metabolite profiling of plasma and urine samples has been completed and data analysis is currently underway.

**Table 1:** Basic characteristics of the KarMeN study participants.

Characteristics of participants	Total (N=301)	Women (n=129)	Men (n=172)
Postmenopausal women, n	73	73	—
Age (years), mean (SD)	47.5 (17.1)	51.7 (15.0)	44.4 (17.9)
Age (years), range	19-80	19-80	20-80
Height (cm), mean SD	174.4 (9.5)	166.8 (6.5)	180.1 (7.2)
Body weight (kg), mean (SD)	72.9 (12.0)	64.4 (8.3)	79.2 (10.2)
Body mass index (kg/m^2^), mean (SD)	23.9 (2.9)	23.2 (2.9)	24.4 (2.7)
Waist circumference (cm), mean (SD)	84.1 (9.7)	79.1 (8.3)	87.8 (8.9)

## Discussion

Our main objective was to analyze the human metabolome in blood and in urine by targeted and untargeted metabolite profiling (GC×GC-MS, GC-MS, LC-MS/MS, and^1^H NMR spectroscopy) and to determine the impact of sex, age, body composition, diet, and physical activity on metabolite profiles of healthy women and men. Therefore, we performed the KarMeN study and established a strictly scheduled experimental setting with a high degree of experimental standardization. SOPs have been developed and applied on recruitment, examinations, and also on the preanalytical handling of samples. All participants underwent the same procedures according to a specified timeline and all samples were treated identically. This contributed to minimizing variations related to examination, sampling, and data analysis. Because blood and urine samples were taken under defined conditions within a scheduled narrow time frame, the influence of circadian variation [26-29] was minimized. We expanded standardization to issues of data collection, sample preparation, and storage because these also have been reported to be factors of variation [41-46]. In addition to these technical aspects, we aimed to comprehensively characterize the study participants to identify metabolite profiles related to age, sex, body composition, diet, physical activity, fitness, and others. Only healthy nonsmoking male and female participants were included in the study according to our strict inclusion and exclusion criteria and after physical examination. Despite the known influence of smoking on the metabolome [32], this information is often not provided. Participants with acute or chronic diseases or on medication were excluded because we targeted healthy individuals. Associations of distinct metabolite patterns and diseases, such as cardiovascular disease [1-3], cancer [4-7], and others [8,9], have been extensively described and are not the topic of our study. Participant characterization also included a broad set of clinical and physiological examinations covering anthropometry, body composition by DXA, pulmonary function, ECG, blood pressure, and arterial stiffness, which have not been applied in metabolomics studies of healthy subjects so far. An additional strength of our study is the unique assessment of current and long-term diet, as well as current and regular physical activity and cardiorespiratory fitness in this cohort of healthy men and women. The scheduled timing of examinations and sampling allowed us to differentiate between current and long-term effects of diet as well as physical activity on metabolite profiles, while enabling us at the same time to consider confounders such as age and sex in the KarMeN study.

So far, most studies reported data from either plasma/serum [47-49] or from urine [50,51]. In this study, we determined the human metabolome in blood and in urine. Further, we applied a combined targeted and untargeted multiplatform metabolite profiling (GC×GC-MS, GC-MS, LC-MS/MS, and^1^H NMR spectroscopy) for each study participant using only one sample set [48]. This multiplatform approach allows identifying a broader spectrum of metabolites compared to a single-platform metabolomics approach. The cross-sectional design of the KarMeN study does not allow conclusions on metabolite variations over longer periods of time. This is one limitation of our study. Additionally, we did not include smokers and healthy obese individuals. Therefore, results of the KarMeN study cannot be transferred to the general population.

## Abbreviations

DXA: dual-energy x-ray absorptiometry
FSH: follicle-stimulating hormone
GC: gas chromatography
KarMeN: Karlsruhe Metabolomics and Nutrition
LC: liquid chromatography
MS: mass spectrometry
NMR: nuclear magnetic resonance
PABA: para-aminobenzoic acid
QC: quality control
SOP: standard operation procedure
